# Biodiversity inventory of the grey mullets (Actinopterygii: Mugilidae) of the Indo‐Australian Archipelago through the iterative use of DNA‐based species delimitation and specimen assignment methods

**DOI:** 10.1111/eva.12926

**Published:** 2020-02-11

**Authors:** Erwan Delrieu‐Trottin, Jean‐Dominique Durand, Gino Limmon, Tedjo Sukmono, Kadarusman, Hagi Yulia Sugeha, Wei‐Jen Chen, Frédéric Busson, Philippe Borsa, Hadi Dahruddin, Sopian Sauri, Yuli Fitriana, Mochamad Syamsul Arifin Zein, Régis Hocdé, Laurent Pouyaud, Philippe Keith, Daisy Wowor, Dirk Steinke, Robert Hanner, Nicolas Hubert

**Affiliations:** ^1^ UMR 5554 ISEM (IRD, UM, CNRS, EPHE) Université de Montpellier Montpellier Cedex France; ^2^ Museum für Naturkunde Leibniz Institute for Evolution and Biodiversity Science Berlin Germany; ^3^ UMR 9190 MARBEC (IRD, UM, CNRS, IFREMER) Université de Montpellier Montpellier Cedex France; ^4^ Maritime and Marine Science Center of Excellence Universitas Pattimura Ambon Indonesia; ^5^ Department of Biology Universitas Jambi Jambi Indonesia; ^6^ Politeknik Kelautan dan Perikanan Sorong Kota Sorong Indonesia; ^7^ Research Center for Oceanography Indonesian Institute of Sciences Jakarta Indonesia; ^8^ Institute of Oceanography National Taiwan University Taipei Taiwan; ^9^ UMR 7208 BOREA (MNHN, CNRS, UPMC, IRD, UCBN) Muséum National d’Histoire Naturelle Paris Cedex France; ^10^ UMR 250 ENTROPIE (IRD, UR, UNC, CNRS, IFREMER), Centre IRD‐Occitanie Montpellier France; ^11^ Division of Zoology Research Center for Biology Indonesian Institute of Sciences (LIPI) Cibinong Indonesia; ^12^ Centre for Biodiversity Genomics University of Guelph Guelph ON Canada; ^13^ Department of Integrative Biology University of Guelph Guelph ON Canada

**Keywords:** Coral Triangle, cryptic diversity, DNA barcoding, reference library, taxonomic gap

## Abstract

DNA barcoding opens new perspectives on the way we document biodiversity. Initially proposed to circumvent the limits of morphological characters to assign unknown individuals to known species, DNA barcoding has been used in a wide array of studies where collecting species identity constitutes a crucial step. The assignment of unknowns to knowns assumes that species are already well identified and delineated, making the assignment performed reliable. Here, we used DNA‐based species delimitation and specimen assignment methods iteratively to tackle the inventory of the Indo‐Australian Archipelago grey mullets, a notorious case of taxonomic complexity that requires DNA‐based identification methods considering that traditional morphological identifications are usually not repeatable and sequence mislabeling is common in international sequence repositories. We first revisited a DNA barcode reference library available at the global scale for Mugilidae through different DNA‐based species delimitation methods to produce a robust consensus scheme of species delineation. We then used this curated library to assign unknown specimens collected throughout the Indo‐Australian Archipelago to known species. A second iteration of OTU delimitation and specimen assignment was then performed. We show the benefits of using species delimitation and specimen assignment methods iteratively to improve the accuracy of specimen identification and propose a workflow to do so.

Box 1 I was given the opportunity to stay for 2 years at Louis’s lab just after my PhD. I arrived at Laval University on a post‐doc contract with the task to handle the DNA barcoding campaign of the Canadian freshwater fishes. T he task was ambitious with a tight schedule – sampling in a year, publication during the second year ‐ and expectations were high as the stake was to showcase the effectiveness of DNA barcoding for future applications in DNA‐based biomonitoring of Canada freshwater fishes. After publishing the results of the campaign in 2008, and now that 11 years have passed since then, I realize how much this experience structured my own scientific thinking and future career and helped me grow. Despite the challenge ahead at that time, sampling 200 species in a country as vast as Canada, Louis has advised, encouraged and facilitated and all this with the relax attitude that Louis is known for. Thanks to Louis I have had the opportunity to interact with a wide community of fish geneticists throughout the country and start collaborating with folks at the University of Guelph such as Bob Hanner, Dirk Steinke, Alex Borisenko, Sujeevan Ratnasingham, Merdad Hajibabei, Natalia Ivanova, Evgeny Zakharov and of course Paul Hébert. Thanks to Louis I had the chance to meet many valuables peoples that largely influenced my career later and with whom I am still collaborating (see this study). So, thanks Louis for the tremendous opportunity you gave me 14 years ago and happy birthdays! Nicolas Hubert.

## 1 INTRODUCTION

DNA‐based methods for species discovery and specimen identification, most notably DNA barcoding (Hebert, Ratnasingham, & deWaard, 2003; Hebert, Stoeckle, Zemlak, & Francis, 2004), offer unprecedented levels of resolution of biological complexity and open new perspectives for the inventory of life on earth. Based on the use of the cytochrome oxidase 1 (COI) as an internal species tag for metazoans, DNA barcoding was proposed as a standardized method for assigning unknown individuals to known species (Floyd, Abebe, Papert, & Blaxter, 2002; Hebert, Cywinska, Ball, & deWaard, 2003), relying on the assumptions that species boundaries had been previously recognized and that DNA barcodes can aptly capture them (Hubert & Hanner, 2015). Very quickly, DNA barcoding has also integrated routines of biodiversity inventories for automated species delimitation (Butcher, Smith, Sharkey, & Quicke, 2012; Janzen et al., 2005; Riedel, Sagata, Suhardjono, Tänzler, & Balke, 2013; Smith et al., 2008; Tänzler, Sagata, Surbakti, Balke, & Riedel, 2012). While this application was not the initial aim of DNA barcoding, it has been suggested that aside from specimen identification, biodiversity inventory may benefit from a universal molecular method of species delineation (Hajibabaei, Singer, Hebert, & Hickey, 2007; Hebert & Gregory, 2005).

At the core of all DNA barcoding initiatives lies the construction and validation of reference libraries that can be further used to assign unknown specimens to known species (Hubert et al., 2008; Ratnasingham & Hebert, 2007). However, reference libraries available in public repositories such as BOLD or GenBank can host a substantial portion of taxonomic misidentifications (Bridge, Roberts, Spooner, & Panchal, 2003; Vilgalys, 2003) leading to ambiguous identifications at the species level (Ardura, Planes, & Garcia‐Vazquez, 2013; Bortolus, 2008). Several recent studies have evidenced that our taxonomic knowledge of living organism is still limited, with the presence of cryptic diversity and/or conflicting taxonomic hypotheses of species delineation, limiting the implementation of automated molecular identifications (Delrieu‐Trottin et al., 2019; Dettai et al., 2017; Hebert, Penton, Burns, Janzen, & Hallwachs, 2004; Hubert et al., 2012; Kadarusman et al., 2012; Meyer & Paulay, 2005; Smith, Wood, Janzen, Hallwachs, & Hebert, 2007; Winterbottom, Hanner, Burridge, & Zur, 2014). These conflicting cases have promoted the use of DNA barcoding for the sorting of species through DNA‐based methods (Butcher et al., 2012; Fujiwasa & Barraclough,^2013^; Hajibabaei et al., 2007; Hebert & Gregory, 2005; Kapli et al., 2017; Puillandre, Modica, et al., 2012; Ratnasingham & Hebert, 2013; Riedel et al., 2013; Tänzler et al., 2012; Zhang, Kapli, Pavlidis, & Stamatakis, 2013). The use of DNA barcoding for both species delineation and specimen identification, however, resulted in some controversies about the objectives and limits of DNA barcoding (Desalle, Egan, & Siddall, 2005; Ebach & Holdredge, 2005; Will, Mishler, & Wheeler, 2005). Species delimitation and specimen identification do not rely on the same theoretical framework, and a few studies have suggested that an iterative use of both is potentially beneficial when it comes to building and maintaining reference libraries (Hubert, Delrieu‐Trottin, Irisson, Meyer, & Planes, 2010; Hubert & Hanner, 2015).

We here exemplify the benefits of the iterative use of species delimitation and specimen identification methods based on a carefully crafted DNA barcode library used as a test case with the fish family Mugilidae (grey mullets) in the Indo‐Australian Archipelago (Durand, Hubert, Shen, & Borsa, 2017). This fish family illustrates the stakes associated with inventorying complex and diverse groups with difficult taxonomy and systematics (Durand & Borsa, 2015; Durand et al., 2012). Currently scattered across 30 genera distributed at a global scale (Froese & Pauly, 2019), the 78 recognized species show strikingly conserved morphological attributes, making species identification challenging (Thomson, 1997). As a consequence, grey mullets are often under‐represented in field guides and specimen identifications are usually extremely difficult for most nonspecialists. Grey mullets, however, constitute a valuable source of protein and income for local communities in many tropical countries through either artisanal fisheries or aquaculture (Bacheler, Wong, & Buckel, 2005; Crosetti & Blaber, 2016; Whitfield, Panfili, & Durand, 2012).

So far, 21 species have been reported from the Indo‐Australian Archipelago (Froese & Pauly, 2019) that varyingly appear in field guides (Kottelat, Whitten, Kartikasari, and Wirjoatmodjo (1993): 16 species; Allen and Erdmann (2012): 4 species; Kottelat (2013): 25 species; White et al. (2013): 7 species) and recent molecular surveys detected substantial levels of cryptic diversity, suggesting that mugilid diversity is severely underestimated (Durand & Borsa, 2015; Durand et al., 2017). This likely accounts for the large amount of mislabeled mugilid sequences in international DNA sequence repositories (Durand et al., 2017), making them of limited use for the identification of unknown specimens through automated engines.

In the present study, we aim to demonstrate the benefits of an iterative use of species delimitation and specimen identification methods to tackle the inventory of a complex taxonomic group, the Indo‐Australian mugilids. We first re‐examined a publicly available and curated DNA barcode reference library for Mugilidae across their distribution range (827 sequences, Durand et al., 2017) through four species delimitation methods to produce a robust scheme of species delimitation and identify operational taxonomic units (OTUs). We further used this carefully crafted DNA barcode library for the assignment of 245 new DNA barcode records to the species level. In order to detect potential new OTUs or the impact of incomplete coverage of the coalescent trees of each OTUs, the four species delimitation methods were applied to the entire dataset consisting of 1,072 DNA barcodes. In case new OTUs were detected or OTU delimitation was revised, an updated reference library was build including representative sequences of each new OTU. A second specimen assignment analysis was further performed using this updated library. Using an iterative procedure of re‐examination through species delimitation and specimen assignment methods on the whole dataset (1,072 sequences), we generated a fit‐for‐use reference library and quantified the impact of incomplete sampling on specimen assignment. The benefits of iteratively using DNA‐based species delimitation and specimen assignment methods are discussed.

## 2 MATERIALS AND METHODS

### 2.1 DNA barcode reference library

The baseline reference library used in this study originates from Durand et al. (2017). This library consists of 827 DNA barcode records trimmed to 538 base pairs representing 102 known taxa (Table S1). Mugilidae systematics follows that of Durand et al. (2012) and Xia, Durand, and Fu (2016) established on the basis of multi‐locus molecular phylogenies. Species nomenclature follows that of Durand and Borsa (2015).

### 2.2 Sampling, sequencing, and data repository

**Figure 1:**
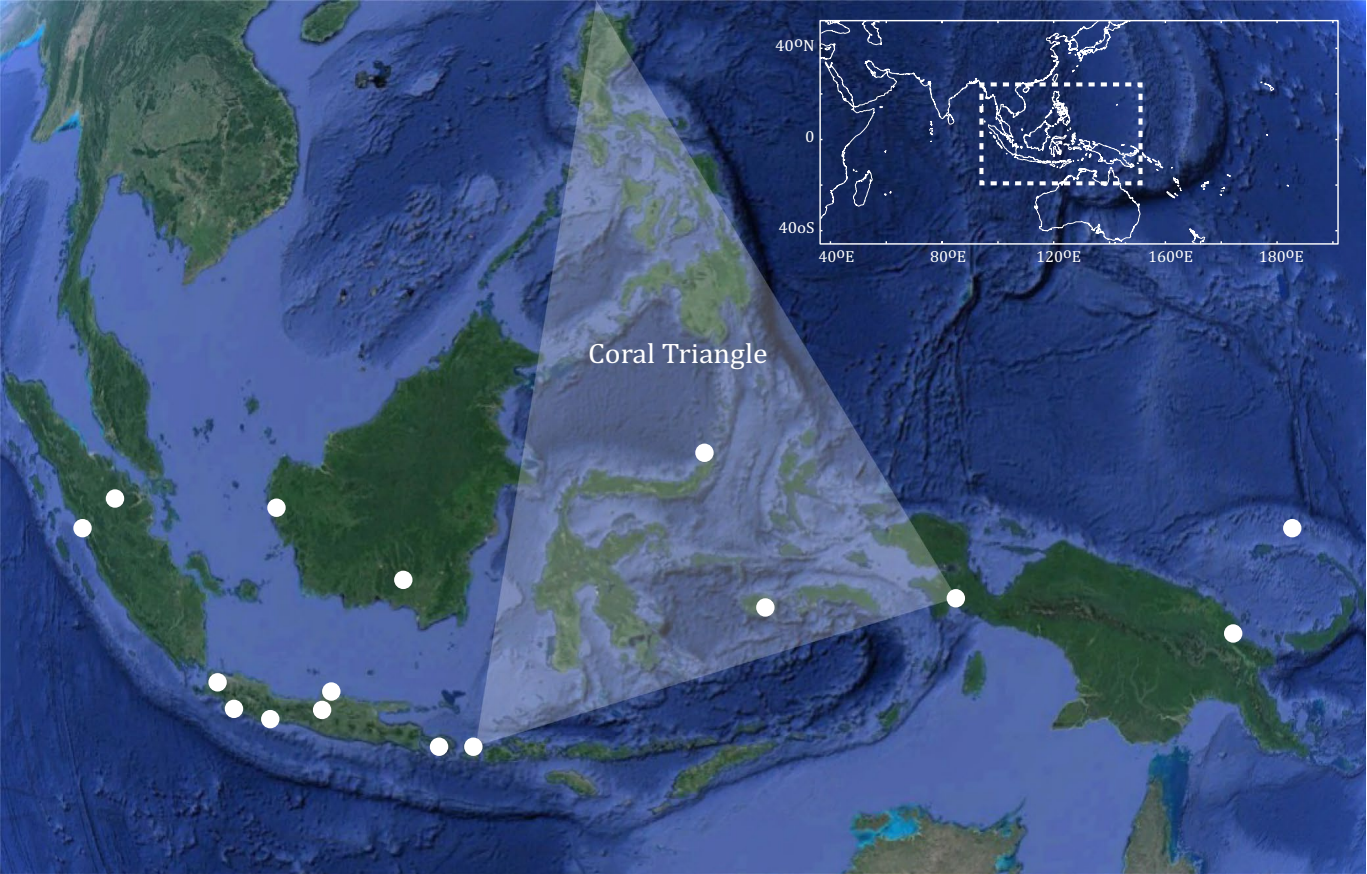
Collection sites for the 245 individuals collected and analyzed in the present study. Each point may represent several collection sites

A total of 245 specimens were collected by visiting fish markets and scuba diving using polespears or captured using various gears including seine nets, cast nets, and gill nets across 25 sites in the Indo‐Australian Archipelago (23 sites in Indonesia and 2 sites in Papua New Guinea, Figure 1). Specimens were photographed and individually labeled, and voucher specimens were preserved in a 5% formalin solution or a 70% ethanol solution. A fin clip or a muscle biopsy was taken for each specimen and fixed in a 96% ethanol solution for further genetic analyses. Both tissues and voucher specimens were deposited in the National Collections at the Research Centre for Biology (RCB) and Research Centre for Oceanography (RCO) from the Indonesian Institute of Sciences (LIPI).

Genomic DNA was extracted for all specimens using a Qiagen DNeasy 96 tissue extraction kit following the manufacturer's specifications. A 651‐bp segment from the 5′ region of the cytochrome oxidase I gene (COI) was amplified using primers cocktails C_FishF1t1/C_FishR1t1 including a M13 tails (Ivanova, Zemlak, Hanner, & Hebert, 2007). PCR amplifications were done on a Veriti 96‐well Fast (ABI—Applied Biosystems) thermocycler with a final volume of 10.0 μl containing 5.0 μl Buffer 2×, 3.3 μl ultrapure water, 1.0 μl each primer (10 μM), 0.2 μl enzyme Phire^®^ Hot Start II DNA polymerase (5 U), and 0.5 μl of DNA template (~50 ng). Amplifications were conducted as follows: initial denaturation at 98°C for 5 min followed by 30 cycles of denaturation at 98°C for 5 s, annealing at 56°C for 20 s, and extension at 72°C for 30 s, followed by a final extension step at 72°C for 5 min. The PCR products were purified with ExoSap‐IT^®^ (USB Corporation) and sequenced in both directions. Sequencing reactions were performed using the “BigDye^®^ Terminator v3.1 Cycle Sequencing Ready Reaction,” and sequencing was performed on an automatic sequencer ABI 3130 DNA Analyzer (Applied Biosystems). The sequences and collateral information (photographs, voucher collection number, and collection data) are publicly available in BOLD (Ratnasingham & Hebert, 2007) and are available in the projects BIFV and WPRFM (Table S2) and as a dataset (https://doi.org/10.5883/DS-BIFMU). DNA sequences were submitted to GenBank; accession numbers are accessible directly at the individual records in BOLD.

### 2.3 Genetic distances, OTU delimitations, and specimen assignments

Kimura 2‐parameter (K2P; Kimura, 1980) pairwise genetic distances were calculated using the R package Ape 4.1 (Paradis, Claude, & Strimmer, 2004). Maximum intraspecific and nearest neighbor genetic distances were calculated from the matrix of pairwise K2P genetic distances using the R package Spider 1.5 (Brown et al., 2012). We checked for the presence of a barcoding gap, that is, the lack of overlap between the distributions of the maximum intraspecific and the nearest neighbor genetic distances (Meyer & Paulay, 2005), by plotting both distances and examining their relationships on an individual basis instead of comparing both distributions independently (Blagoev et al., 2016). A neighbor‐joining (NJ) tree based on K2P distances was built using ape 4.1 to visually inspect genetic distances and DNA barcode clusters (Figure S1).

For the sake of clarity, species identified based on morphological characters are referred to as species while species delimited by DNA sequences are referred to operational taxonomic unit (OTU), defined as diagnosable molecular lineages (Avise, 1989; Moritz, 1994; Vogler & DeSalle, 1994). OTUs were delimitated using four different algorithms: (a) Refined Single Linkage (RESL) as implemented in BOLD and used to produce Barcode Index Numbers (BINs; Ratnasingham & Hebert, 2013), (b) Automatic Barcode Gap Discovery (ABGD; Puillandre, Lambert, Brouillet, & Achaz, 2012), (c) Poisson Tree Process (PTP) in its multiple rate version (mPTP) as implemented in the stand‐alone software mptp_0.2.3 (Kapli et al., 2017; Zhang et al., 2013), and (d) General Mixed Yule‐Coalescent (GMYC) in its multiple rate version (mGMYC) as implemented in the R package Splits 1.0‐19 (Fujisawa & Barraclough, 2013). Both RESL and ABGD used DNA alignments as input, while a maximum likelihood (ML) tree was used for mPTP and a Bayesian chronogram was reconstructed for mGMYC based on a strict‐clock model using a 1.2% of genetic distance per million years (Bermingham, McCafferty, & Martin, 1997). The ML tree for mPTP algorithm was generated with RAxML (Stamatakis, 2014) based on a GTR + Γ substitution model. The ultrametric and fully resolved tree for mGMYC was reconstructed using the Bayesian approach implemented in BEAST 2.4.8 (Bouckaert et al., 2014). Two Markov chains of 50 million each were run independently using a Yule pure birth model tree prior, a strict‐clock model, and a GTR + I + Γ substitution model. Trees were sampled every 10,000 states after an initial burn‐in period of 10 millions, both runs were combined using LogCombiner 2.4.8, and the maximum credibility tree was constructed using TreeAnnotator 2.4.7 (Bouckaert et al., 2014). Duplicated sequences were pruned prior to the Bayesian analysis.

Three specimen assignment methods implemented in the R package BarcodingR version 1.0.2 (Zhang, Hao, Yang, & Shi, 2017) in R 3.4.0 (R Core Team, 2017) were used to assign unknown specimen to known species: (a) the back‐propagation neural networks method **(**BP), a machine learning approach inferring species membership using DNA sequence data based on a neural network algorithm (Zhang, Sikes, Muster,& Li, 2008; Zhang & Savolainen, 2009); (b) the fuzzy set‐based approach method (FZ), a distance method based on a K‐nearest neighbor (KNN) search algorithm with a fuzzy membership function to estimate the probability of a query sequence belonging to the nearest neighbor reference DNA barcode sequence (Zhang, Muster, et al., 2012); and (c) the alignment‐free kmer‐based method (FZKMER), suitable for both coding and noncoding portions of the genomes using machine learning (Zhang, Feng, et al., 2012) as the optimal kmer length is first estimated followed by an FZ specimen identification. We included one representative sequence, randomly selected, for each OTU of the reference libraries to perform the assignments. We used the *barcoding.spe.identify* function specifying “*bpNewTrainingOnly”* in the first run and “bpUseTrained” in the second run of the function to perform BP. We used the *barcoding.spe.identify* function with the option “fuzzyId” to perform FZ and the *barcoding.spe.identify2* function specifying a search for a kmer length up to 5 to perform the FZKMER method. Both FZ and FZKMER methods assign to each potential identification an “FMF value” in the range of 0–1, indicating likelihood of the assignment (Zhang, Feng, et al., 2012), while BP assigns to each identification a “bp.probilities” also in a range of 0–1. For the sake of clarity, we will refer hereafter to both the bp.probabilities and the FMF values as “probabilities.” As the different methods can lead to conflicting results, a consensus has been established for the multiple methods using the *consensus.identify* function of the barcodingR package. We considered that a consensus emerged if at least two methods were converging. Finally, we computed the ratio of the intraspecific K2P genetic distance of a selected OTU to the nearest neighbor K2P genetic distance for each method to test whether the selected OTU was the least distant possible and spot potential false positives (i.e., specimens incorrectly assigned).

The delimitation and the assignment methods were used iteratively: Species delimitation methods were first applied to the 827 DNA barcode reference library, and specimen assignment methods applied to the 245 newly generated DNA barcodes. A second round of species delimitation was performed on the whole dataset, that is, the 827 DNA barcode reference library and the 245 newly generated DNA barcodes (1,072 sequences), in order to check for the consistency of the delimitation schemes. We added one representative sequence of each new OTU retrieved and removed/added the corresponding sequences when OTUs were merged/split following this second iteration of delimitation to perform a second round of specimen assignment of the newly generated DNA barcodes. The comparison of the two rounds of species delimitations and specimen assignments also allowed us to appraise the impact of the taxonomic coverage of the reference library on the accuracy of the specimen identifications, that is, whether OTU attributed by specimen assignment methods corresponds to the OTU given by the species delimitation methods, and to evaluate the behavior of the probabilities associated with the true (correctly assigned) and false (erroneously assigned) positives.

## 3 RESULTS

### 3.1 First round of species delimitation—identifying OTUs in the published reference library

**Figure 2:**
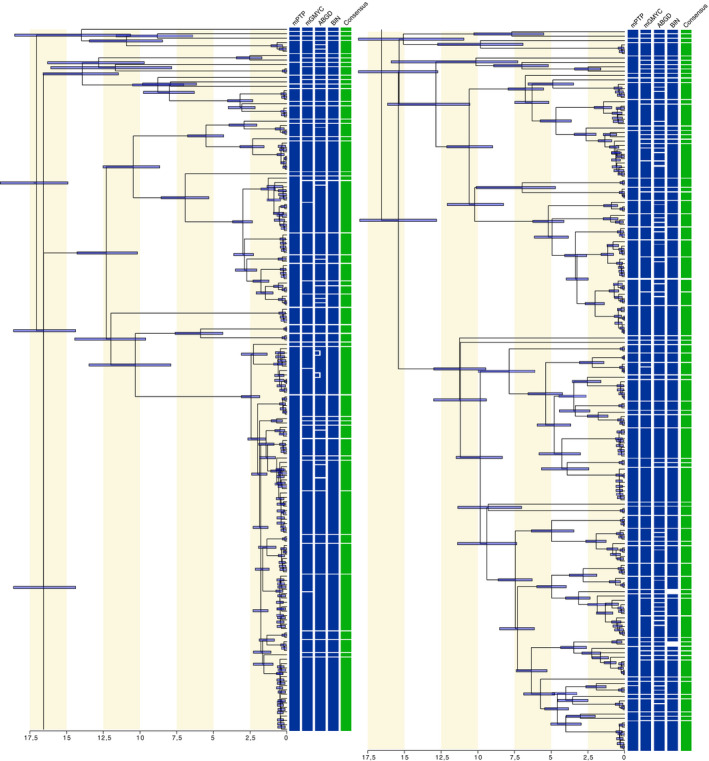
Bayesian maximum credibility tree of the 326 unique haplotypes of the 827 DNA barcode reference library (round 1 of species delimitation) including 95% HPD intervals for node age estimates and OTU delimitation schemes according to the four species delimitation methods implemented (blue) and the resulting consensus (green)

The first round of species delimitation methods using the DNA barcode reference library of Durand et al. (2017) composed of 827 sequences and 102 nominal species yielded a varying number of OTUs according to the methods with 105 using RESL, 148 using ABGD, 70 using mPTP, and 120 using mGMYC (Figure 2 and Table S3) and resulting in a consensus consisting of 113 OTUs (Figure 2). Such discrepancies between the estimated number of OTUs and the observed number of nominal species were due to a substantial number of cryptic lineages (i.e., morphologically undistinguishable OTUs) observed within *Crenimugil* sp. A (OTUs 36 and 37), *Mugil curema* (OTUs 74 and 75), *Mugil* sp. O (OTUs 70 and 73), *Osteomugil perusii* (OTUs 43, 97, 98, and 99), *Osteomugil* sp. (OTUs 41, 45, 100)*; Planiliza* sp. (OTUs 18, 24, 27, and 103), *Planiliza* sp. B (OTUs 104 and 105)*, Planiliza* sp. H. (OTUs 29 and 30), *Planiliza subviridis* (OTUs 16, 17, and 113), and *Mugil* sp. (OTUs 68 and 96). A single instance of species pair indistinguishable by the species delimitation methods was observed (*Mugil* sp. N and *Mugil margaritae*, OTU 72). Finally, several cases of conflicting grouping were detected. Some sequences of *Dajaus* sp. B clustered with *Dajaus monticola* (OTU 60); one sequence of *Chelon ramado* clustered with *Chelon auratus* (OTU 5); one sequence of *Mugil* sp. M clustered with *Mugil curema* (OTU 74); and one sequence of *Planiliza* sp. H clustered with *Planiliza macrolepis* (OTU 28).

**Figure 3:**
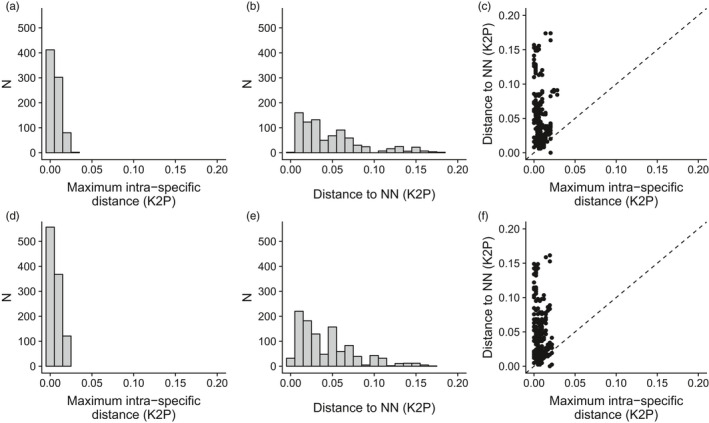
Distribution of genetic distance for the first (top) and second (bottom) DNA barcode reference libraries of this study. (a, d) Distribution of the maximum intraspecific genetic distances (K2P, percent); (b, e) Distribution of the nearest neighbor genetic distances (K2P, percent); (c, f) Relationship between maximum intraspecific and nearest neighbor genetic distances. Points above the diagonal line indicate OTUs with a barcode gap

The maximum K2P intraspecific distances ranged from 0.00000 to 0.02828 (Figure 3a), while the nearest neighbor distances ranged from 0.00000 to 0.18403 (Figure 3b). The median nearest neighbor distance (0.03478) was 8.76‐fold higher than the median intraspecific distance (0.00397; Figure 3c). A single lineage (OTU 90) displayed a lower nearest neighbor K2P distance than its maximum intraspecific distance. Finally, nearest neighbor K2P distances below one percent of pairwise genetic distance were observed for 5 species, with 2 species displaying a K2P genetic distance of 0 to their nearest phylogenetic relative (OTU 16, OTU 113)*.*

### 3.2 First round of specimen assignment—assigning new unknown specimen to known species

**Figure 4:**
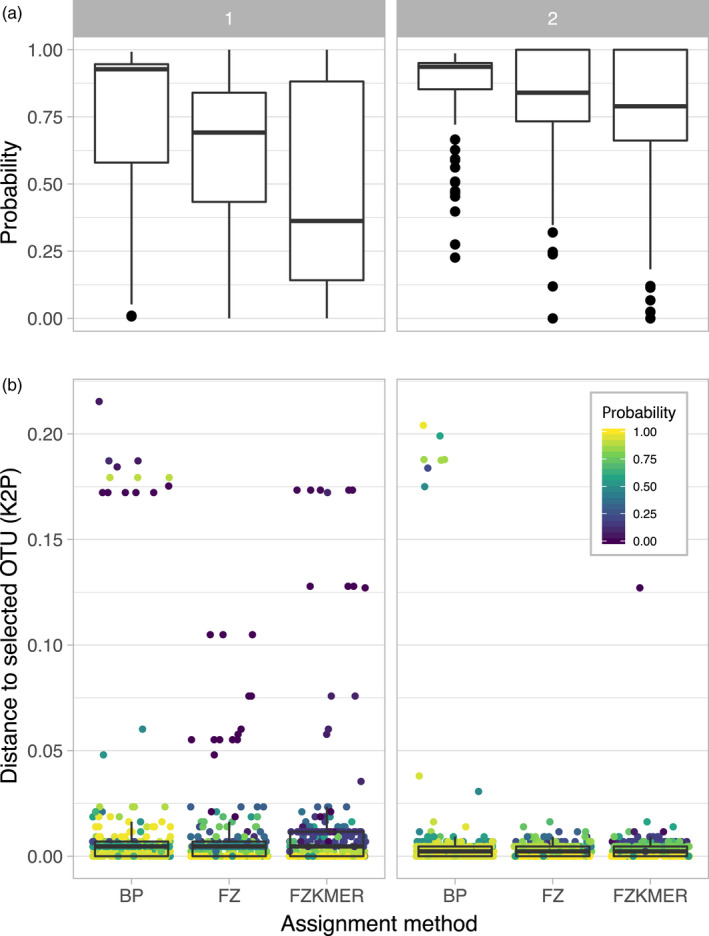
Results of the first round of specimen assignment. (a) Distribution of probabilities associated with the three assignment methods for the first (1) and the second (2) iteration of specimen assignment; and (b) Distance to selected OTU for the different assignment methods for both iteration of species assignment (first at the left side, second at the right side). The lower and the upper hinges of the box plots correspond to the first and the third quartiles; the lower whiskers correspond to the smallest and observation greater than or equal to lower hinge − 1.5 × interquartile range, while upper whiskers correspond to the largest observation less than or equal to upper hinge + 1.5 × interquartile range

**Figure 5:**
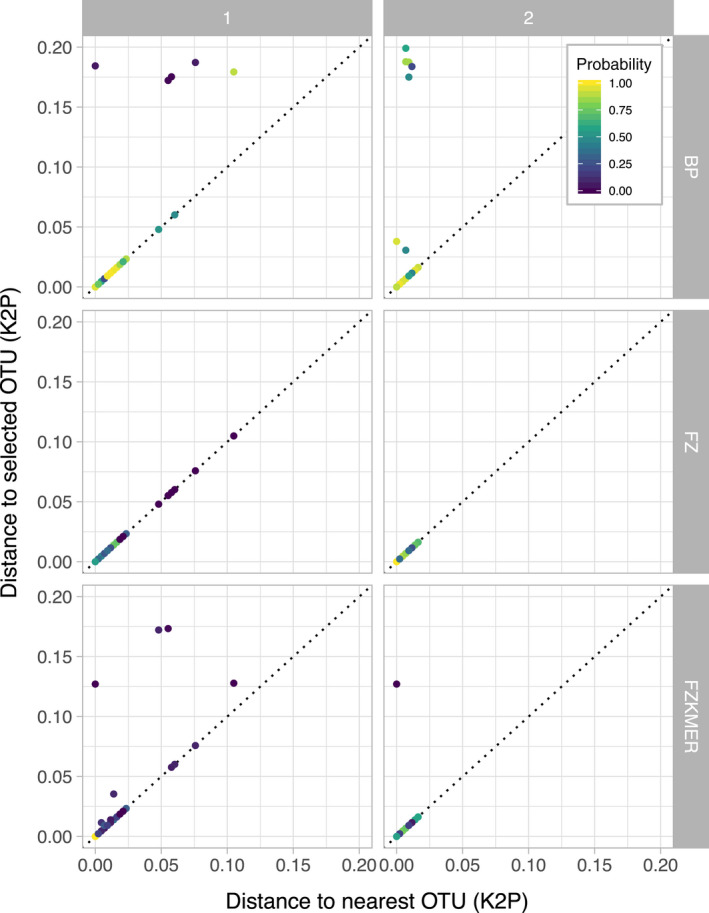
K2P genetic distances to the selected OTUs and K2P genetic distances to the nearest OTUs for the different specimen assignment methods for the first round (1) and second round (2) of specimen assignments

**Figure 6:**
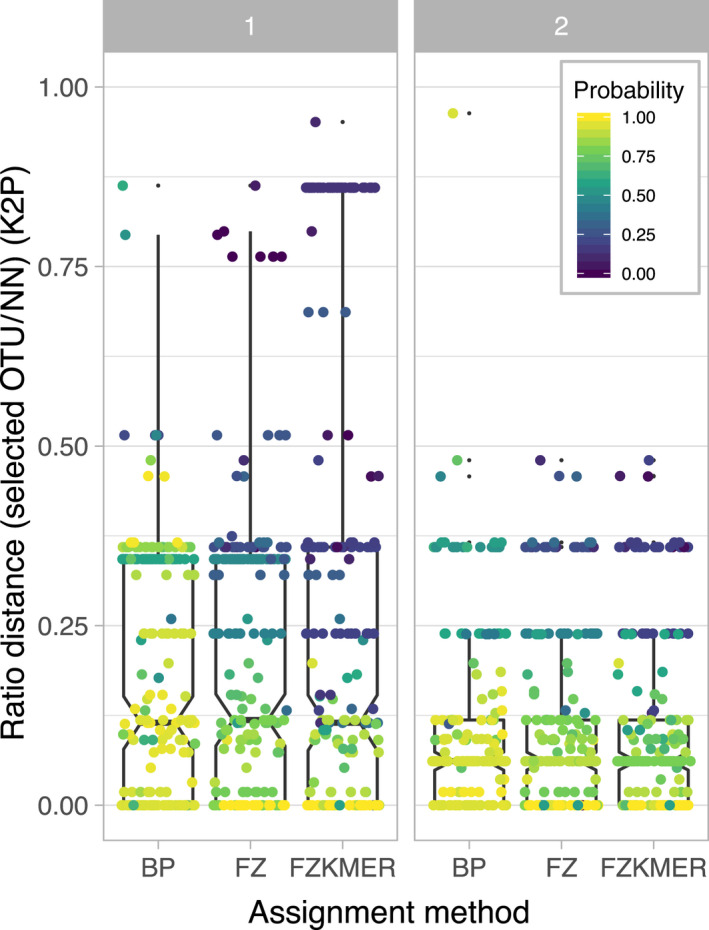
Distribution of the specimen assignment probabilities across the ratio of the K2P genetic distances to the selected OTU upon the K2P genetic distances to the nearest neighbor in the reference library (selected OTU/ NN) for each of the specimen assignment methods for the first round (1) and second round (2) of specimen assignment

**Figure 7:**
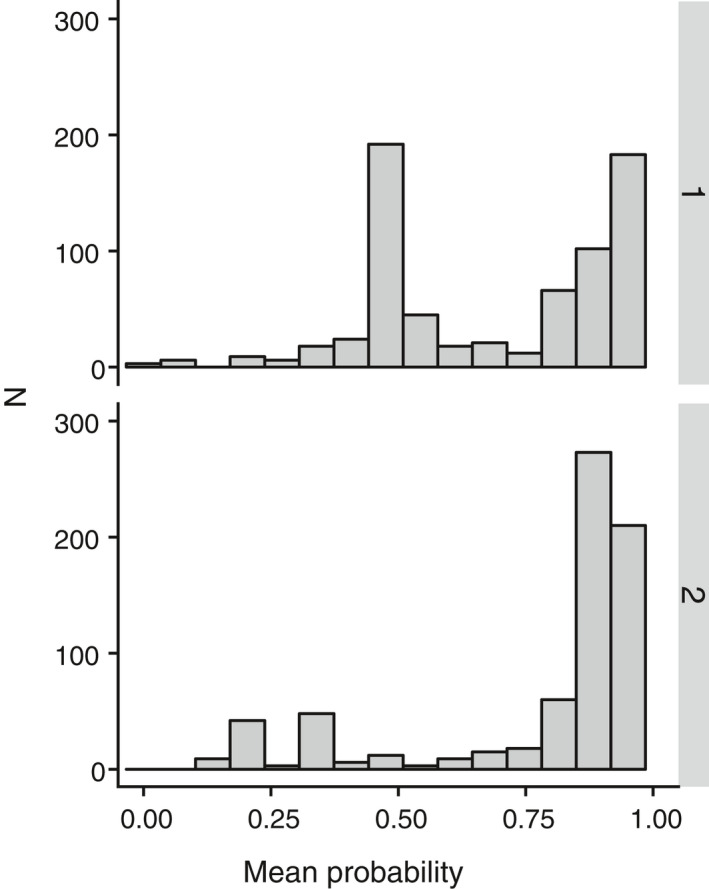
Distribution of the average probability of the three assignment methods for each specimen assigned during the first round (1) and second round (2) of specimen assignment

All these sequences were above 500 bp, and no stop codons were detected. Probabilities associated with each specimen assignment varied significantly between the three methods, χ^2^(39) = 167.19, *p* < .001, with BP showing higher probabilities (median: 0.92) than the two other methods (FZ median: 0.74; FZKMER median: 0.57; Figure 4a and Table S3). We also compared the probabilities associated with each assignment method to the distance to the assigned OTU (Figure 4b). Both the FZ and FZMER methods systematically attributed probabilities values lower than 0.5 for sequences displaying a distance to the selected OTU higher than 0.015 (Figure 4b). In 16 cases, BP and FZKMER assigned more distantly related OTUs than the nearest OTU available in the library while FZ did this in 8 cases (Figure 5). Finally, the distribution of the ratio of the K2P genetic distance to the selected OTUs to the K2P genetic distance to the nearest neighbor is highly similar among all methods (Figure 6), but noticeable differences are observed in the probabilities provided by each assignment method. For instance, BP attributed probabilities close to 1 to assignments with a ratio of up to 0.45 while FZ and FZKMER display probabilities lower than 0.25 at such ratio levels. Finally, despite these differences between the three methods, they were generally in agreement regarding the species assigned; a consensus emerged for most cases (95.9%) with the three methods of assignment converging in 77.1% of the cases and 2 out of the 3 methods converging in 18.8% (Table S3). The median probability associated with such consensus was over 0.77 (Figure 7), while the mean probability associated with cases where no consensus emerged (*n* = 10) was 0.20.

### 3.3 Second round of species delimitation—revising OTU delimitation by incorporating the new unknown specimens

The second round of species delimitation applied to the joint dataset of 1,072 DNA barcodes yielded more OTUs in general with 113 using RESL, 161 using ABGD, 82 using mPTP, and 110 using mGMYC (vs. 105, 148, 70, and 120 for the first round; Figure S1 and Table S3) with a consensus consisting of 121 OTUs, that is eight additional OTUs (OTUs 96, 105, 116 to 121) compared to the first round of species delimitation. A few OTUs observed during the first round of delimitation were merged during the second round (OTUs 90 and 96, and OTUs 33 and 39). In addition, this second round of delimitation showed that the 245 unknown specimens belong to 27 different OTUs (Table S4) corresponding to 12 known OTUs described in Durand et al. (2017) including OTU 25 (*Planiliza melinoptera*), OTU 29 (*Planiliza* sp. H), OTU 34 (*Crenimugil buchanani*), OTU 35 (*Crenimugil* sp. D), OTU 36 (*Crenimugil* sp. A), OTU 47 (*Ellochelon vaigensis*), OTU 48 (*Plicomugil labiosus*), OTU 104 (*Planiliza* sp. B), OTU 109 (*Planiliza* sp. G), OTU 114 (*Crenimugil* sp. C), OTU 115 (*Crenimugil* sp. B), and OTU 120 (*Planiliza* sp. E); seven that results from an alternative scheme of delimitation compared to Durand et al. (2017) including OTUs 22 and 107 for *Planiliza* sp. D; OTUs 43, 98, and 99 for *O. perusii;* and OTUs 113 and 119 for *P. subviridis*; and eight new taxa that are observed for the first time in the present study including OTU 27 (*Planiliza* sp.), OTU 41 (*Osteomugil* sp.), OTU 96 (*Ellochelon* sp.), OTU 105 (*Crenimugil* sp.), OTU 116 (*Crenimugil* sp.), OTU 117 (*Osteomugil* sp.), OTU 118 (*Osteomugil* sp.), and OTU 121 (*Planiliza* sp.).

The maximum K2P intraspecific distances calculated for the 1,072 sequences ranged from 0.00000 to 0.021795, while the nearest K2P neighbor distance ranged from 0.00000 to 0.17064. The median nearest neighbor distance was lower (0.03180) here than in the first round of OTU delimitation (0.03478) while the median intraspecific distance (0.004762) was higher (0.00397), leading to a lower ratio (6.68) of those two values compared to the first round (8.76). In contrast to the first round of species delimitation, seven lineages (OTUs 16, 22, 115, 87, 89, 90, and 115) now display lower nearest neighbor K2P distances than their maximum intraspecific distance. Finally, nearest neighbor K2P pairwise distances below 1 percent were observed for eight OTUs with two of them displaying a K2P distance of 0 to their nearest phylogenetic relative (again OTUs 16 and 113)*.*

Comparing the results of the first round of specimen assignment with the second round of species delimitation showed that only 213 out of the 245 new sequences (86.9%) had been correctly assigned during the first assignment stage, with a mean probability of 0.72 (±0.23), while the mean probability for false positives was 0.39 (±0.24). BP is attributing relatively similar probabilities to true positives (0.88 ± 0.11) and false positives (0.72 ± 0.36), while both FZ and FZKMER delivered distinct mean probabilities for true positives (respectively, 0.68 ± 0.28 and 0.60 ± 0.38) and false positives (respectively, 0.29 ± 0.32 and 0.16 ± 0.16). It is worth noting that most of these 32 false positives correspond to the new OTUs detected during the second delimitation round and that no consensus could be found for a third of them.

### 3.4 Second round of specimen assignment—final assignment of unknowns to knowns

**Figure 8:**
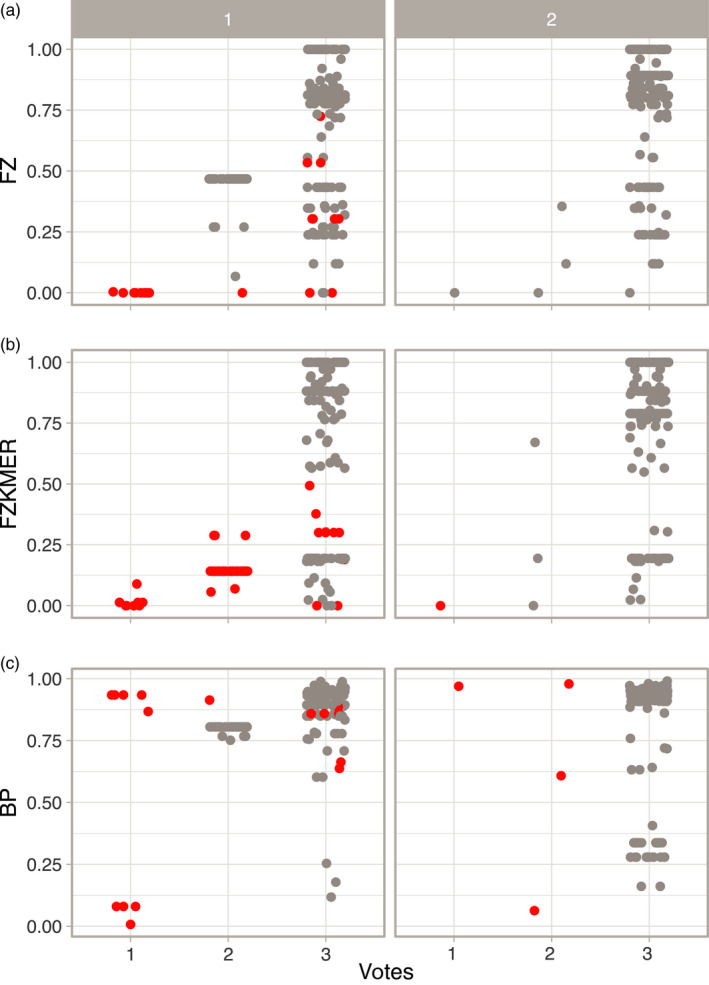
Specimen assignment probabilities for each specimen assignment method for the first round (1) and second round (2) of specimen assignment, with false positives in red and true positives in gray

Similarly to the first round of specimen assignment, the BP method attributed higher probabilities than the two other methods. Yet, a shift toward higher probabilities was observed for all three methods, with a median value of 0.94 for BP, 0.86 for FZ, and 0.84 for FZKMER. In comparison with the first round, the range of distances to selected OTUs is smaller for all three methods with the FZ now being the only method with distances to selected OTUs not larger than 0.020 (Figure 4b). FZ always selected the nearest OTU, while both BP and FZKMER still assigned several sequences to OTUs more distantly related than the nearest OTU available in the library (eight and one sequences respectively; Figure 5). Interestingly, all these nine cases correspond to misidentifications (assignment to an OTU different from the OTU attributed by the delimitation methods) while only two out of 40 cases reported for the first assignment round corresponded to misidentifications. The remaining 38 cases represented not yet delineated new OTUs. The ratio between the K2P genetic distances of the selected OTUs and distance to the nearest neighbor was smaller for the three methods in the second round (Figure 6). A consensus between the 3 assignment methods emerged in 99.5% of the cases, with the three methods converging in this second round for 97% of the cases and 2 out of the 3 methods converging in 2.5% (Table S3). The median probability associated with a consensus was over 0.87 (Figure 7). Finally, we evaluated the potential presence of false positives comparing assignments made by the three different methods with the species delimitation consensus. With BP, seven false positives (97% of correct ID) were retrieved with probabilities ranging from 0.06 to 0.98 (Figure 8c) while FZKMER delivered only one false positive with a probability of 0 (99.6% of correct ID; Figure 8b) and FZ provided only true positives (Figure 8a). A consensus emerged for all but one case; we found no false positives among those consensuses and the only case where no consensus emerged (ambiguous ID); FZ was the only method assigning the sequence to the correct OTU. Finally, the mean probability associated with true positives is larger than 0.74 for all 3 methods (mean value of 0.85 ± 0.22 for BP; 0.78 ± 0.35 for FZ; and 0.75 ± 0.29 for FZKMER).

**Figure 9:**
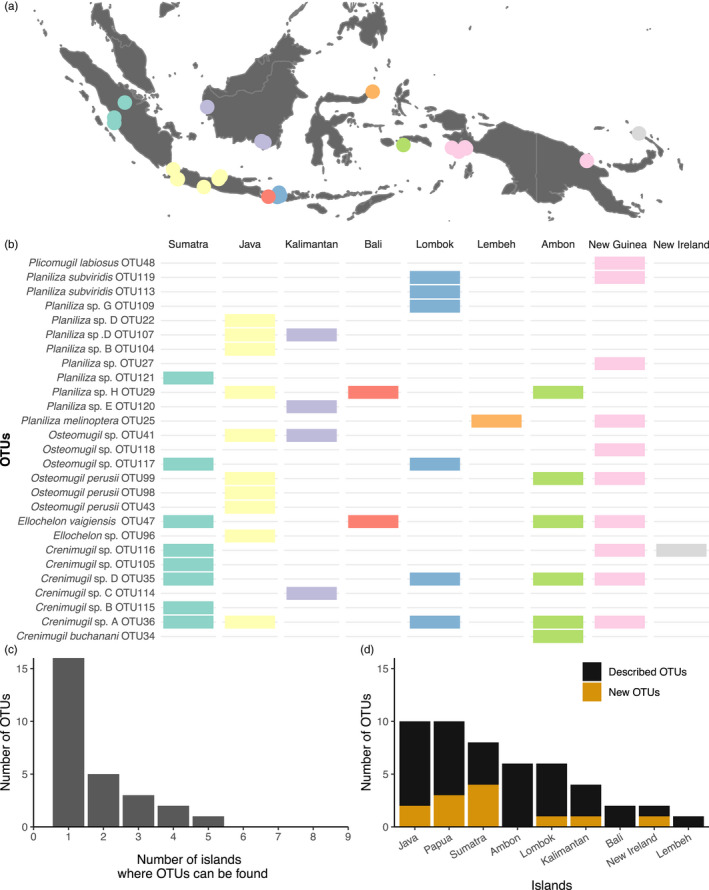
(a, b) Geographic distribution across the IAA of the 27 OTUs retrieved among the 245 individual analyzed, each dot on the map representing a collection site; (c) distribution of the OTU diversity as a function of the number of islands where they occur and (d) distribution of OTU richness per island

The second round of specimen assignment assigned the 237 new unknown specimens (sequences of 8 specimens were used as representatives of new OTUs) to 27 OTUs with varying distribution ranges (Figure 9). More than half of the OTUs have been found in only one island: five in Java (OTU 22 (*Planiliza* sp. D)*,* OTU 43 (*O. perusii*), OTU 96 (*Ellochelon* sp.), OTU 98 (*O. perusii*), and OTU 104 (*Planiliza* sp. B)), three in Sumatra (OTU 105 (*Crenimugil* sp.), OTU 115 (*Crenimugil* sp. B), and OTU 121 (*Planiliza* sp.)), three in New Guinea (OTU 118 (*Osteomugil* sp.), OTU 48 (*P. labiosus*), and OTU 27 (*Planiliza* sp.)), two in Kalimantan (OTU 114 (*Crenimugil* sp. C) and OTU 120 (*Planiliza* sp. E)), two in Lombok (OTU 109 (*Planiliza* sp. G) and OTU 113 (*Planiliza subviridis*)), and one in Ambon (OTU 34 (*C. buchanani*)), while OTU 36 (*Crenimugil* sp. A) has been collected in five different islands, from Sumatra to New Guinea (Figure 9a–c). Finally, at the island level, the number of OTUs collected ranged from one (Lembeh) to 10 (Java and New Guinea). No new taxa were collected from Ambon, Bali, and Lembeh, while the other islands hosted up to four new taxa (Sumatra; Figure 9d).

## 4 DISCUSSION

DNA‐based automated specimen identification methods, such as DNA barcoding, open new perspectives to inventory, and monitor biodiversity (Deiner et al., 2017). In the last fifteen years, the success of the DNA barcoding initiative has given rise to the development of multiple approaches to not only assign unknown specimens to known species (see review of Bazinet & Cummings, 2012) but also to automatically delineate species through DNA‐based approaches (Brown et al., 2012; Munch, Boomsma, Willerslev, & Nielsen, 2008; Munch, Boomsma, Huelsenbeck, Willerslev, & Nielsen, 2008; Pons et al., 2006; Puillandre, Lambert, et al., 2012; Ratnasingham & Hebert, 2013). The present study highlights the benefits of using species delimitation and specimen identification methods jointly and iteratively. Our test case was a family of shore fishes that are among the most complex for morphological species identification and which is plagued by major taxonomic gaps. The first iteration of species delimitation applied to the reference library revealed the presence of more than 10% cryptic diversity because 113 OTUs were extracted from 102 known taxa. However, mitonuclear discordances due to incomplete lineage sorting and introgression are known to limit the robustness of mtDNA species delimitation (Hinojosa et al., 2019; Pedraza‐Marrón et al., 2019; but see review of Toews & Brelsford, 2012). In that case, the use of multiple independent nuclear markers, biparentally inherited, should be used to confirm the mtDNA‐derived cryptic lineages detected here (Fennessy et al., 2016; Fišer, Robinson, & Malard, 2018). Despite using a reference library with objectively delineated OTUs for a first round of specimen assignment, the second iteration of species delimitation showed that 14% of 245 consensus identifications made during the first iteration of specimen assignment were false positives, most of which actually corresponded to new OTUs.

The iterative use of species delimitation and specimen identification methods also resulted in a substantial decrease in the proportion of false positives and a shift toward higher probabilities values for true positives. The resulting delimitation scheme during the second iteration benefitted from an increase in the taxonomic coverage and the number of sequences per OTU in the reference library. This can potentially increase intraspecific genetic diversity, which in turn may help to better delineate OTUs (Kekkonen & Hebert, 2014; Kekkonen, Mutanen, Kaila, Nieminen, & Hebert, 2015). We observed such improvement with the addition of 245 new DNA barcodes as it resulted in the delineation of several new OTUs and the merging of a few OTUs generated during the first round. Furthermore, the increasing number of OTUs resulted in a shift toward a lower range of intraspecific genetic diversity with the effect that the number of false positives dropped substantially for all three specimen assignment methods**—**with no more false positives being observed for consensus assignment. Such results call for an iterative use of species delimitation and specimen assignment methods, especially when confronted with taxonomically complex groups such as observed here.

The present study also clearly emphasizes the importance of developing comprehensive reference libraries to overcome the susceptibility of specimen assignment methods to spurious OTU delimitation resulting from insufficient taxonomic and/or intraspecific genetic diversity coverage. The accuracy of the specimen assignment procedure, that is used to assign an unknown specimen to a known species (Hubert et al., 2008), relies both on OTU/species coverage and on accuracy of OTU boundaries in the reference library. The ubiquity of sequencing with the emergence of new genomic tools led to the generation of thousands of uncurated sequences in international repositories. The lack of curation and the ongoing difficulty to identify species of a large portion of the Tree of Life have led to a large amount of misidentified records, Mugilidae being no exception (Durand et al., 2017). Yet, most specimen assignment methods require a well‐parameterized reference library, locally or globally accessible. Not all international repositories, unlike BOLD, have been conceived to handle taxonomic updates in the long term, a task that requires extensive collateral data (Ratnasingham & Hebert, 2007; Ward, Hanner, & Hebert, 2009). Ideally, local, limited, and self‐generated DNA barcode reference libraries should be the starting point as they allow to perform a variety of species delimitation and specimen identification methods, but at the same time provide control over the accuracy of the identifications (DiBattista et al., 2017; Olds et al., 2016; Sonstebo et al., 2010; Willerslev et al., 2014).

The combined application of several species delimitation methods allows for the normalization of over‐ or underestimation that can occur with each of these methods (Blair & Bryson, 2017; Huang et al., 2018; Kekkonen & Hebert, 2014; Kekkonen et al., 2015). Similarly, the possibility to compute different specimen identification methods within the same framework allows for the improvement of the confidence in the inferences by application of consensus methods. In fact, specimen assignment methods will always assign a unknown specimen to a known species, with a certain level of confidence, leading potentially false positives. Despite using a well‐curated library, we showed that the issue of false positive could not be put aside.

We suggest two approaches to improve the accuracy of specimen assignment and to avoid false positive identifications. The first approach is to use probability thresholds, and the second approach involves the use of several assignment methods to establish a consensus. After our first assignment round, we observed the presence of false positives for all three methods. Yet, computing a consensus showed that a third of the false positives appeared as "ambiguous identification." Moreover, probabilities associated with false positive were no larger than 0.5 and 0.75 for FZKMER and FZ, respectively, when all three methods converged toward the same identity. BP was the method generating most false positives among the three methods investigated in this study and can be considered as the least reliable for the present dataset. FZ method was the only method that did not display any false positives after the second round of species delimitation irrespective of whether the identification was supported by the two other methods or not. This result is in agreement with the recommendation of Zhang et al. (2017) to the use of the FZ method to avoid potential false positives in cases of incomplete taxon coverage in a reference library.

Finally, the current Mugilidae checklist for the Indo‐Australian Archipelago was far from being established as demonstrated by the large range of species numbers found in the literature for this family (Kottelat et al. (1993): 16 species; Allen and Erdmann (2012): 4 species; Kottelat (2013): 25 species; Shen and Durand (2016): 29 species). We found that the 245 specimens studied here belonged to 27 OTUs spreading across five different genera. Less than half of these OTUs correspond to known species, cryptic diversity was found for three of these species, hosting up to three different lineages, and eight (30%) potentially new species have been collected (i.e., unknown and newly detected), enlightening the importance of integrative approaches to disclose hidden diversity (Hebert, Penton, et al., 2004; Janzen et al., 2005; Smith et al., 2008, 2007). Such results represent a new example of the benefits of using the DNA barcoding standards in taxonomy (Butcher et al., 2012; Hubert et al., 2008; Ratnasingham & Hebert, 2007, 2013) especially when confronted to such complex group and advocate toward covering the largest area possible to depict the diversity of species and complex of species. Indeed, our starting reference library (Durand et al., 2017) contained no specimens from the Indo‐Australian archipelago despite a rather large taxonomic coverage. The occurrence of these new OTUs after adding specimens from these regions is not surprising given they are located in the Coral Triangle (CT) region, a region characterized by a large still not fully explored diversity of shore fishes (Allen & Erdmann, 2012). To date, no endemic species of grey mullets have been described in this region so far; our results suggest that regional species richness of grey mullets is underestimated. The very restricted distributions of these endemic species among the IAA call for a thorough investigation of the underlying mechanism that led to such pattern, making the mullets a great candidate to investigate the origin of endemism in the IAA region (Connolly, Bellwood, & Hughes, 2003; Hughes, Bellwood, & Connolly, 2002; Mora et al., 2003; Reaka, Rodgers, & Kudla, 2008). As mullets represent a target for local communities in this region, such high proportion of very restricted endemic species retrieved here is also of importance for conservation program.

## 5 CONCLUSION

DNA barcoding has prompted the development of a wide range of genomic tools. As concerning the use of bad taxonomy can be in ecology (Bortolus, 2008), the use of incomplete reference libraries can have dramatic consequences too. We demonstrated the benefits of working with curated libraries and proposed a workflow to minimize the effect of incomplete sampling on specimen identifications, proposing the iterative use of species delimitation and specimen assignment methods. This iterative approach showed that despite extensive effort to clarify the taxonomy of Mugilidae (Durand & Borsa, 2015; Durand et al., 2017; Shen & Durand, 2016; Xia et al., 2016), cryptic diversity can still be found in this group. Our inventory of the biodiversity of grey mullets in the IAA region also led to the discovery for the first time of a large portion of endemic species of mugilids with very restricted range size, a result of importance in term of both conservation and evolutionary process.

## CONFLICT OF INTEREST

None declared.

## Data Availability

These sequence and collateral data have been deposited in BOLD (projects BIFV and WPRFM) and are publicly available as a dataset (BIFMU, https://doi.org/10.5883/DS-BIFMU). DNA sequences have also been submitted to GenBank; accession numbers are accessible directly at the individual records in BOLD. R script to perform the assignments used in the manuscript is available on GitHub https://github.com/edelrieutrottin/Assigning_Grey_mullets and Zenodo https://doi.org/10.5281/zenodo.35433425.
