# Supplementary material for: Biodiversity inventory of the grey mullets (Actinopterygii: Mugilidae) of the Indo‐Australian Archipelago through the iterative use of DNA‐based species delimitation and specimen assignment methods
Source: Evol Appl. 2020 Feb 11;13(6):1451–67. doi: 10.1111/eva.12926 (PMC7359824; doi:10.1111/eva.12926)

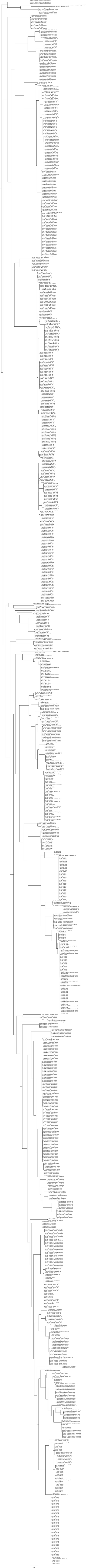

Supplement: Supplementary file 1 [file EVA-13-1451-s001.pdf]
